# RNA Virus-Encoded miRNAs: Current Insights and Future Challenges

**DOI:** 10.3389/fmicb.2021.679210

**Published:** 2021-06-24

**Authors:** Asuka Nanbo, Wakako Furuyama, Zhen Lin

**Affiliations:** ^1^Molecular and Cellular Virology, Research Center for the Control and Prevention of Infectious Diseases, Nagasaki University, Nagasaki, Japan; ^2^Tulane University Health Sciences Center and Tulane Cancer Center, New Orleans, LA, United States

**Keywords:** RNA virus, microRNA, coding RNA, pathogenesis, replication, immunoevasion, therapeutics

## Abstract

MicroRNAs are small non-coding RNAs that regulate eukaryotic gene expression at the post-transcriptional level and affect a wide range of biological processes. Over the past two decades, numerous virus-encoded miRNAs have been identified. Some of them are crucial for viral replication, whereas others can help immune evasion. Recent sequencing-based bioinformatics methods have helped identify many novel miRNAs, which are encoded by RNA viruses. Unlike the well-characterized DNA virus-encoded miRNAs, the role of RNA virus-encoded miRNAs remains controversial. In this review, we first describe the current knowledge of miRNAs encoded by various RNA viruses, including newly emerging viruses. Next, we discuss how RNA virus-encoded miRNAs might facilitate viral replication, immunoevasion, and persistence in their hosts. Last, we briefly discuss the challenges in the experimental methodologies and potential applications of miRNAs for diagnosis and therapeutics.

## Introduction

MicroRNAs (miRNAs) are small non-coding RNAs with approximately 15−25 nucleotides in length, which mediate post-transcriptional gene silencing by binding to complement sequences in the target mRNAs. Since the discovery of the first miRNA, *lin-4*, in *Caenorhabditis elegans* (Lee et al., 1993; Wightman et al., 1993), numerous miRNAs have been identified in diverse eukaryotic species. The involvement of miRNAs in various physiological processes, such as cell proliferation, differentiation, cell death, and disease progression, has been well documented (Bartel, 2004;2018). Shortly after the discovery of cellular miRNAs, the first virus-encoded miRNAs were identified in Epstein-Barr virus (EBV)-infected cells (Pfeffer et al., 2004). To date, 569 mature viral miRNAs have been identified from a variety of viral families and deposited in the miRbase^1^. The majority of identified viral miRNAs are derived from DNA viruses, predominantly in the herpesvirus family, and have been studied in detail for their biogenesis, functional contributions to virus?host interactions, and viral pathogenesis (Skalsky and Cullen, 2010; Grundhoff and Sullivan, 2011; Kincaid and Sullivan, 2012; Bruscella et al., 2017; Cardin and Borchert, 2017). In contrast to miRNAs encoded by DNA viruses, the functional roles of RNA virus-encoded miRNAs (rv-miRNAs) in the virus life cycle remain unclear. One reason for limited knowledges of rv-miRNAs is that RNA viruses carry a smaller genome than DNA viruses, allowing them to have fewer options for encoding miRNAs. Another reason may be that most RNA viruses replicate in the cytoplasm, which prevents access of viral RNA genomes to the miRNA biogenesis machinery in the nucleus. Moreover, processing of miRNAs from the viral genome may lead to genome instability in the virus, and, consequently, may have negative impacts on viral replication. Recently, the advantages of novel sequencing technologies have contributed to the identification of potential non-coding RNAs encoded by diverse RNA viruses; however, their functions have not been well studied. Here, we summarize current insights into miRNAs encoded by different families of RNA viruses, including newly emerging viruses, by focusing on their physiological roles in the viral life cycle and pathogenicity. We also discuss the challenges particularly in the experimental methodologies and potential applications of miRNAs for diagnosis and therapeutics of RNA virus-associated diseases.

## RNA Viruses

Viruses are classified into two main groups, DNA and RNA viruses, depending on their genetic materials. RNA virus can be subdivided into three groups in terms of their genome orientation, such as double-stranded RNA (dsRNA; e.g., *Reoviridae*), single-stranded positive-sense RNA [(+)ssRNA; e.g., *Flaviviridae*], and single-stranded negative-sense RNA [(-)ssRNA; e.g., *Orthomyxoviridae*] viruses. In addition, *Retroviridae* possesses the (+)ssRNA genome that is reverse transcribed into DNA and then integrated into the host genome to establish a persistent infection. Most RNA viruses replicate in the cytoplasm, whereas others including *Orthomyxoviridae* and *Retroviridae* complete transcription and replication of their genomes in the nucleus.

## Biogenesis of miRNAs

**FIGURE 1:**
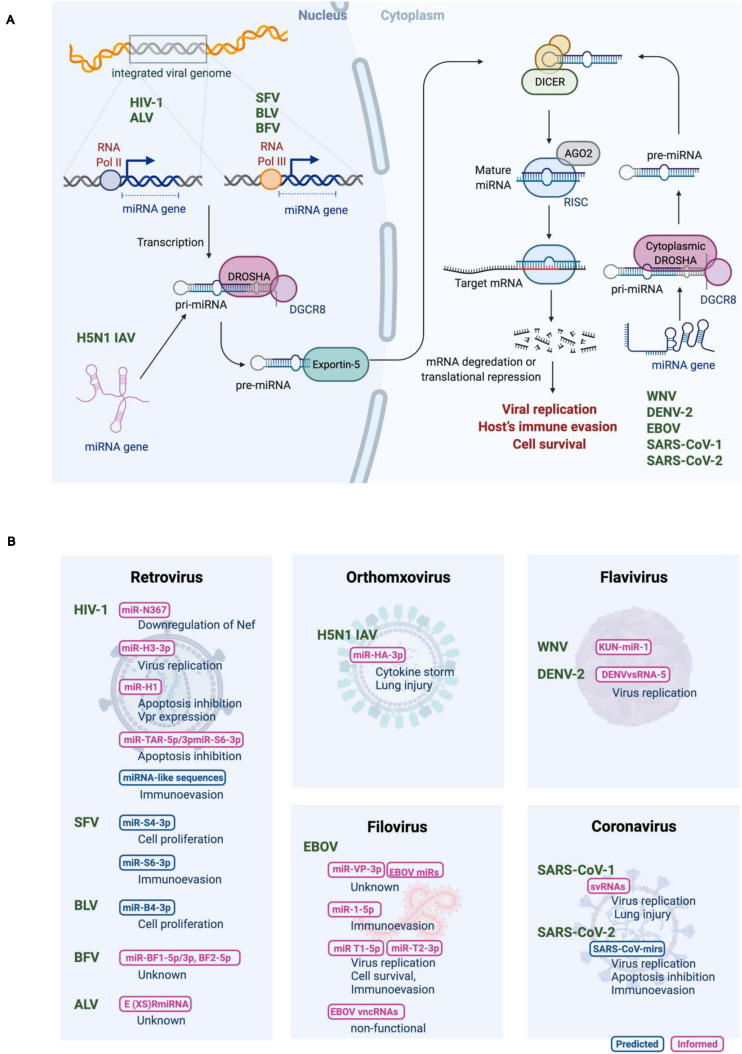
**(A)** Biogenesis of RNA virus encoded micro RNAs (rv-miRNAs). Canonical miRNAs are transcribed by RNA polymerase II (Pol II) as pri-miRNAs from either the protein-coding region or the non-coding region of the viral genome in the nucleus. Viral genomes of the retroviruses are reverse transcribed into DNA, which is then integrated into a host genome. While transcription of rv-miRNAs encoded by human immunodeficiency virus-1 (HIV-1), and avian leukosis virus (ALV) is mediated by Pol II, rv-miRNAs encoded by simian foamy virus (SFV), bovine leukemia virus (BLV) and bovine foamy virus (BFV) are transcribed by RNA polymerase III (Pol III). Viral RNA genome of influenza A virus (IAV) of the H5N1 subtype replicates in the nucleus. Cleavage of pri-miRNAs by Drosha results in pre-miRNA that is exported to the cytoplasm via exportin 5. In the cytoplasm, pre-miRNAs undergo cleavage by Dicer to generate the mature miRNAs that are loaded in the multiprotein RISC complex to target the host mRNA transcript. The mature rv-miRNAs of west Nile virus (WNV), dengue virus 2 (DENV-2), Ebola virus (EBOV), and severe acute respiratory syndrome coronavirus (SARS-CoV) are likely processed by the cytoplasmic Drosha. **(B)** Summary of proposed rv-miRNAs’ functions. Computationally predicted and experimentally evaluated rv-miRNAs are shown in blue or magenta, respectively. Adapted from “miRNA in Cancer,” by BioRender.com (2020). Retrieved from https://app.biorender.com/biorender-templates.

miRNAs are expressed in the cells of higher eukaryotic organisms (Bartel, 2004;2018). Viruses exploit host miRNA biogenesis machinery to produce their own miRNAs (Figure 1). Biogenesis of canonical miRNAs is mainly initiated by RNA polymerase II (Pol II)-mediated transcription from either the protein-coding region or the non-coding region of the genome to generate the primary miRNA transcripts (pri-miRNAs). Pri-miRNAs are usually more than 1−2 kb in length and contain a long stem-loop structure. Pri-miRNAs are then processed in the nucleus to generate ∼60 nucleotide-long precursor miRNA (pre-miRNA) hairpin, which is mediated by a nuclear microprocessor protein complex consisting of a member of the RNase III nuclease superfamily, Drosha, and its cofactor dsRNA-binding protein, DiGeorge syndrome critical region 8 (DGCR8) (Lee et al., 2003; Landthaler et al., 2004). Pre-miRNAs are exported into the cytoplasm by a nuclear export protein exportin-5 (Okada et al., 2009), where they undergo a second round of processing by a RNase III complex DICER, resulting in a ∼22 nucleotide-long dsRNA duplex (Hutvagner et al., 2001). The product contains the guide strand (mature miRNA) and its reverse complement (passenger strand). Even though both strands could be functional, typically the guide strand alone is recruited to the multiprotein RNA-induced silencing complex (RISC), where mature miRNA binds to target RNA, whereas the other strand is degraded (Khvorova et al., 2003; Schwarz et al., 2003). As a key component of the RISC, Argonaute (AGO) supports the interaction between miRNAs and target mRNAs and promotes mRNA decay by recruiting deadenylases and decapping factors onto the target mRNAs through trinucleotide repeat containing 6A (TNRC6) (Hammond et al., 2001). In mammals, target recognition is primarily mediated by complementarity between the miRNA seed regions (positions 2–8 of the mature miRNAs) and miRNA recognition elements in the target mRNAs (Bartel, 2018). Metazoan miRNAs generally target the 3′ UTR of mRNA, where miRNAs induce translational repression and degradation of mRNAs, depending on the number of available binding sites and the degree of base pairing (Bartel, 2004; Baek et al., 2008; Chekulaeva and Filipowicz, 2009).

Most RNA viruses replicate in the cytoplasm, and viral RNAs cannot interact with nuclear miRNA processors, which may be a possible reason for the lack of robust evidence of RNA viruses producing functional miRNAs. Moreover, in particular, (+)ssRNA viruses undergo transcription of the entire genome to form a polyprotein complex. This complex is then proteolytically cleaved to generate functional viral proteins, suggesting that mature miRNAs derived from the viral genome may target their own genome, leading to a negative impact on viral replication. On the contrary, cytoplasmic translocation of Drosha was demonstrated during virus infection, allowing the robust biogenesis of miRNAs in the cytoplasm (Shapiro et al., 2012). Moreover, cytoplasmic RNA viruses, such as tick-borne encephalitis virus and Sindbis virus, which were inserted an exogenous miRNA hairpin, were reported to express functionally mature miRNAs lacking the ability of viral replication and expression of functional viral proteins (Rouha et al., 2010; Shapiro et al., 2010). These data support the model that cytoplasmic RNA viruses employ a certain strategy to circumvent these potential barriers. In fact, several RNA viruses that replicate in the cytoplasm, including west Nile virus (WNV), dengue virus 2 (DENV-2), Ebola virus (EBOV), and severe acute respiratory syndrome coronavirus (SARS-CoV), have been verified to produce functional miRNAs, as discussed further below.

## RNA Virus-Encoded miRNAs (rv-miRNAs)

In this review, we describe the current insights into rv-miRNAs, including their underlying physiological functions involved in the viral life cycle and pathogenesis. To date, rv-miRNAs have been identified in several RNA virus families, such as *Retroviridae*, *Orthomyxoviridae*, *Flaviviridae*, *Filoviridae*, and *Coronaviridae*, as described below. An overview of representative rv-miRNAs encoded by these RNA viruses is summarized in Table 1.

**TABLE 1:** RNA virus-encoded miRNAs.

**Virus family**	**Species**	**miRNAs**	**Identification**	**Proposed Targets**	**Proposed functions**	**References**
*Retroviridae*	HIV-1	miR-N367		NRE in 5’ LTR	Downregulation of Nef	Omoto et al., 2004; Omoto and Fujii, 2005
		miR-H3-3p		TATA box in 5’ LTR	Virus replication	Zhang et al., 2014
		miR-H1		AATF, host miR-140	Apoptosis inhibition, Vpr expression	Kaul et al., 2009
		miR-TAR-5p/3p		Apoptosis-related genes	Apoptosis inhibition	Ouellet et al., 2008; Klase et al., 2009
		microRNA-like sequences		Mimic of host OncomiRs	Immunoevasion	Holland et al., 2013
	SFV	miR-S4-3p		Mimic of host miR-155	Cell proliferation	Kincaid et al., 2014
		miR-S6-3p		Mimic of host miR-132	Immunoevasion	Kincaid et al., 2014
	BLV	miR-B4-3p		Mimic of host miR-29	Cell proliferation	Gillet et al., 2007
	BFV	miR-BF1-5p/3p, BF2-5p		Unknown	Unknown	Whisnant et al., 2014
	ALV-J	E (XSR) miRNA		Unknown	Unknown	Yao et al., 2014
*Orthomuxoviridae*	H5N1 IAV	miR-HA-3p		PCBP2	Cytokine storm, lung injury	Li et al., 2018
*Flaviviridae*	WNV	KUN-miR-1		GATA4	Virus replication	Davis et al., 2007; Hussain et al., 2012
	DENV-2	DENV-vsRNA-5		NS1	Virus replication	Hussain and Asgari, 2014
*Filoviridae*	EBOV	miR-VP-3p		Unknown	Unknown	Chen et al., 2016
		miR-1-5p		Importin-α5	Immunoevasion	Liu et al., 2016
		miR T1-5p, T2-3p		NF-kB, TNF	Virus replication, cell survival, Immunoevasion	Teng et al., 2015
		EBOV miRs		Unknown	Unknown	Liang et al., 2014
		EBOV vncRNAs		Mimic of host miRs	non-functional	Prasad et al., 2020
*Coronaviridae*	SARS-CoV-1	svRNAs		Unknown	Virus replication, lung injury	Morales et al., 2017
	SARS-CoV-2	SARS-CoV-mirs		NF-kB, JAK/STAT, TGF-β	Virus replication, anti-apoptosis, Immunoevasion	Aydemir et al., 2021

*HIV-1: human immunodeficiency virus-1; SFV: simian foamy virus; BLV: bovine leukemia virus; BFV: bovine foamy virus; ALV-J: avian leukosis virus J strain; IAV: Influenza A virus; WNV: west Nile virus; DENV-2: dengue virus 2; EBOV: Ebola virus; SARS-CoV: severe acute respiratory syndrome coronavirus; TAR: the trans-activation responsive; NRE: the negative-response element; LTR: the long terminal repeat; AATF: apoptosis antagonizing transcription factor; PCBP2: Poly(rC)-binding protein 2; GATA4: GATA Binding Protein 4; NS1: the non-structural protein 1; NF-kB: the nuclear factor-kB; TNF: tumor necrosis factor; JAK/STAT: Janus kinase-signal transducer and activator of transcription; TGF-β: transforming growth factor-β.*

### Retrovirus-Encoded miRNAs

Retroviruses are enveloped RNA viruses that reverse transcribe their (+)ss RNA genomes into dsDNA and then integrate into host chromosomes for a persistent infection (Stephen, 2013). To date, the majority of the identified rv-miRNAs are encoded by retroviruses. Several independent groups took advantage of computer-directed analyses to identify putative rv-miRNAs encoded by human immunodeficiency virus (HIV)-1 and predict their potential target transcripts (Bennasser et al., 2004; Pfeffer et al., 2004). The first HIV-1-encoded rv-miRNA, miR-N367, was experimentally identified in virally infected mammalian cells (Omoto et al., 2004). miR-N367 is derived from Nef, the viral immune suppressor factor gene and targets the negative-response element in the 5′ long terminal repeats (LTR) U3 region, which results in downregulation of Nef and subsequent HIV-1 transcription (Omoto and Fujii, 2005). Moreover, exogenous expression of another computationally predicted rv-miRNA, HIV-1-miR-H1 in mammalian cells suppresses the host apoptosis antagonizing transcription factor (AATF) gene and miR-140, leading to apoptosis inhibition and upregulation of the viral accessory protein Vpr, respectively (Kaul et al., 2009). In another study, next generation sequencing (NGS) combined with computational prediction identified miR-H3-3p derived from the reverse transcriptase region of the HIV-1 genome (Zhang et al., 2014). miR-H3-3p is highly conserved among HIV-1 subtypes and targets the TATA box in the 5′ LTR region, leading to increased HIV-1 replication. However, Lin et al. reported contradictory results, showing that HIV-1 lacks the ability to produce rv-miRNAs in infected T cells (Lin and Cullen, 2007). Moreover, some fraction of the studies considers only bioinformatic identification and lacks experimental evaluation of bona fide rv-miRNAs and their targets. For example, Holland et al. demonstrated that the viral Pol and Env-coding regions of the HIV-1 genome generate miRNA-like fragments, which are homologous with several host miRNAs, some of which are cancer-associated miRNAs (oncomiRs) (Holland et al., 2013).

The trans-activation responsive (TAR) element, which forms a stable miRNA precursor-like hairpin structure of ∼50 nucleotides in length, is expressed during virus infection and activates HIV-1 gene transcription (Klaver and Berkhout, 1994). It was reported that the TAR motif encodes rv-miRNAs in latently infected and viral particle-producing cells (Ouellet et al., 2008). The TAR-derived rv-miRNA initiates transcriptional silencing at the LTR promoter via chromatin remodeling and even downregulates apoptotic genes (Klase et al., 2009).

Interestingly, TAR-derived rv-miRNAs are detected in exosomes released from HIV-1-infected cells as well as in the serum from HIV-1-positive patients (Narayanan et al., 2013; Bernard et al., 2014), suggesting that exosomal rv-miRNAs could play a role in viral pathogenesis and be considered as potential biomarkers for virus infection.

Computational analyses also identified rv-miRNAs encoded by other retrovirus species, such as simian foamy virus (SFV) and bovine leukemia virus (BLV) (Gillet et al., 2007; Kincaid et al., 2014). Unlike canonical miRNAs, the SFV and BLV rv-miRNAs are produced by RNA polymerase III (Pol III), which allows only the sub-genomic small RNAs to be cleaved into miRNAs. SFV is commonly found in diverse non-human primate (NHP) species and establishes a life-long infection without any apparent pathogenicity (Meiering and Linial, 2001). SFV miR-S4-3p mimics a cellular oncomiR miR-155 and stimulates proliferative activity of SFV-infected cells. Similarly, SFV miR-S6-3p was reported to be functionally homologous with the interferon-suppressive cellular miR-132 that is responsible for evasion from innate immunity (Kincaid et al., 2014). BLV is a causative agent for enzootic bovine leukemia, which is the most common neoplastic disease of cattle (Polat et al., 2017). BLV-miR-B4-3p mimics the seed sequence of host miR-29, an miRNA overexpressed in a variety of lymphoproliferative disorders and is associated with B cell tumorigenesis in mice (Han et al., 2010; Santanam et al., 2010), suggesting that rv-miRNA expression may sustain the proliferation of infected cells and play a role in BLV-associated tumorigenesis (Gillet et al., 2007). Nevertheless, these studies only indicate the possible roles of their rv-miRNAs in tumorigenesis, and thus these hypotheses need to be addressed experimentally in the context of viral infection.

Bovine foamy virus (BFV) is highly prevalent in cattle, although no obvious disease is associated with viral infection (Mirzaei et al., 2021). BFV encodes three rv-miRNAs (i.e., miR-BF1-5p, miR-BF1-3p, and miR-BF2-5p) that are initially transcribed by RNA Pol III as a single transcript from the 3′ LTR of the viral genome. It is subsequently cleaved to generate two pre-miRNAs that are then processed to yield three distinct, biologically active miRNAs (Whisnant et al., 2014). According to a sequencing analysis of chronically infected cells, these miRNAs represent >70% of the total small RNAs expressed. This study also confirmed the stable existence of miR-BF1-5p and miR-BF2-5p in peripheral leukocytes of infected calves.

Avian leukosis virus (ALV) belongs to the alpha retrovirus family and causes various types of neoplastic diseases in birds (Payne et al., 1991). A highly pathogenic strain, ALV-J, produces miRNA originating from the exogenous virus-specific region, E element, or XSR, which is responsible for ALV-induced tumorigenicity (Chesters et al., 2006) via canonical miRNA biogenesis machinery (Yao et al., 2014).

### Orthomyxovirus-Encoded miRNAs

Influenza A virus (IAV) is a member of *Orthomyxoviridae* and possesses a (-)ssRNA genome with eight distinct gene segments. IAV replicates and transcribes its genome in the nucleus (Wright et al., 2013). H5N1 is a subtype of IAV, which is highly contagious and pathogenic in birds. H5N1 pathogenicity in human is partly mediated by a systematic inflammatory syndrome involving elevated levels of circulating cytokines and hyperactivation of immune cells (cytokine storm) and lung damage (Watanabe et al., 2012). Deep sequencing of a virally infected cell line identified H5N1-encoded miR-HA-3p, which was named for its coding location in the 3′ arm of the viral hemagglutinin segment (Li et al., 2018). Further investigation demonstrated that miR-HA-3p post-transcriptionally downregulates the Poly(rC)-binding protein 2 (PCBP2). PCBP2 negatively regulates retinoic acid-inducible gene-1 and mitochondrial antiviral signaling (RIG-1/MAVS), which is a highly conserved cytoplasmic viral RNA sensor, and then activates a series of antiviral signaling pathways, including an inflammatory response (You et al., 2009). Strikingly, blocking of miR-HA-3p retains PCBP2 expression and suppresses inflammatory cytokine production upon H5N1 infection *in vivo* and *in vitro*. In addition, lung damage and survival rates of mice are also improved by the same approach, suggesting a potential therapeutic strategy against H5N1 infection based on silencing rv-miRNA (Li et al., 2018).

### Flavivirus-Encoded miRNAs

West Nile virus and DENV are cytoplasmic RNA viruses with a (+)ssRNA genome. Both viruses are mosquito-borne and can cause a range of mild to lethal diseases with substantial public health impacts (Wilder-Smith et al., 2017). WNV- and DENV-encoded miRNAs have been investigated. The 3′ terminal stem-loop of the WNV genome has multiple roles in virus-host interactions and virus replication (Davis et al., 2007). In this highly conserved region, a small miRNA-like RNA, KUN-miR-1, was identified by a bioinformatic approach as the first miRNA encoded by cytoplasmic RNA viruses (Hussain et al., 2012), and facilitates viral replication by targeting a transcription activator, GATA binding protein 4 (GATA4), in both virally infected and miRNA-overexpressing mosquito cell lines. A DENV rv-miRNA, DENV-vsRNA-5, has been identified by deep sequencing in both DENV-2 infected-mosquito and mammalian cells (Hussain and Asgari, 2014). DENV-vsRNA-5 suppresses viral replication by targeting the coding region of viral non-structural protein 1 (NS1) of viral genome, suggesting the possible utilization of the small RNA to limit DENV replication.

### Filovirus-Encoded miRNAs

Ebola virus is a (-)ss RNA virus, which causes the severe and often fatal Ebola virus disease (EVD) in humans and NHP as reviewed in Goeijenbier et al. (2014). EBOV has been reported to encode putative pre-miRNA and mature miRNAs by use of computational analysis (Chen et al., 2007; Liang et al., 2014; Teng et al., 2015; Liu et al., 2016; Hsu et al., 2018). Further studies proved exogenous expression of these miRNAs in cell lines (Liang et al., 2014; Teng et al., 2015; Liu et al., 2016). By use of bioinformatics prediction, these rv-miRNAs appear to contribute to viral replication, cell survival, and immune response evasion by targeting multiple host factors (Teng et al., 2015; Liu et al., 2016). Moreover, several studies have detected these miRNAs in patients from an outbreak in West Africa and experimentally infected NHP using NGS, qRT-PCR, and northern blot (Chen et al., 2016; Duy et al., 2018). MiR-VP-3p, which is derived from a viral VP40-coding region, was detected in the exosomal fraction in the serum of EVD patients (Duy et al., 2018). Interestingly, miR-VP-3p is detected during the acute phase but not in the recovery phase in patient serum. Moreover, miR-VP-3p is detectable in the serum of patients before the EBOV genomic RNA becomes positive, suggesting its potential contribution to early diagnosis of EVD. A more recent work identified multiple rv-miRNAs in bat and human cell lines infected by both EBOV and a closely related Marburg virus using deep sequencing and qRT-PCR. These miRNAs are generated in a host miRNA biogenesis machinery-independent manner (Prasad et al., 2020). Moreover, this study demonstrated that two of the identified rv-miRNAs closely match computationally identified RNAs in previous studies (Prasad et al., 2020). However, they lack the ability to induce gene silencing and the suppression of viral replication by acting as antiviral siRNAs, highlighting the current pitfalls of computational identification methods (Prasad et al., 2020).

### Coronavirus-Encoded miRNAs

Severe acute respiratory syndrome coronavirus and SARS-CoV-2 cause respiratory or intestinal infections in humans and other animals and are responsible for the SARS epidemic in 2003 and the currently ongoing COVID-19 pandemic, respectively (Perlman and Netland, 2009; Hu et al., 2021). Morales et al. (2017) identified three rv-miRNAs in lung derived from SARS-CoV-infected mice using deep sequencing. This study further demonstrates that sv-RNA molecules derived from viral nucleoprotein and non-structural protein 3 regions contribute to SARS-CoV pathogenesis, which is reduced by antagonization of these sv-RNAs (Morales et al., 2017). Another bioinformatics-driven prediction study revealed that SARS-CoV-2 encodes multiple putative rv-miRNAs from different regions of the viral genome and targets various signaling molecules involved in apoptosis, immune function, cell cycle, and transcription (Aydemir et al., 2021). However, further investigations are required to experimentally evaluate their expression and biological functions in the context of viral infection.

## Future Challenges

In the past few years, several lines of evidence have demonstrated that RNA viruses encode miRNAs. However, the physiological significance of these rv-miRNAs in viral life cycles and pathogenesis remains largely unclear and warrants further investigation. The reliability of the reported data is limited by the low expression levels of some rv-miRNAs as well as the available detection methods. Although recently developed comprehensive computational prediction has significantly contributed to the identification of rv-miRNAs with low expression levels, currently available algorithms are still suboptimal and often produce a significant number of false predictions (Pinzon et al., 2017). Therefore, a comprehensive approach composed of both computational and experimental methodologies in the context of viral infection is required to further validate the existing data.

A few rv-miRNAs are incorporated into exosomes and stably exist extracellularly in the serum and plasma of EBOV and HIV-1-infected patients (Narayanan et al., 2013; Bernard et al., 2014; Duy et al., 2018). It is widely known that exosomes transfer incorporated miRNAs to remote tissues and organs, where they play a crucial role in various physiological and pathological processes (Zhang et al., 2015). This suggests that extracellular rv-miRNA may also participate in systematic symptoms caused by RNA viral infection.

As evident from the latest outbreaks, emerging RNA viruses have posed an increasingly serious threat to public health. Due to lack of availability of therapeutic treatments against most of these emerging viruses, miRNA-based therapeutics are considered a new class of drugs with potential applications for various diseases including infectious diseases (Chakraborty et al., 2017; Titze-De-Almeida et al., 2017). A major barrier to the application of miRNA-based therapies is the non-toxic delivery of sufficient amounts of miRNAs to infected sites. As host miRNAs are involved in diverse cellular functions, long-term treatment with their antagomir may cause side-effects correlated with deregulation in target gene expression. Therefore, silencing viral miRNAs could be a more reasonable approach in some cases. Further investigation is urgently needed not only to enhance our knowledge of rv-miRNA but also to provide new therapeutically relevant insights.

## Author Contributions

AN contributed to the conceptualization, drafting, and revising the manuscript. WF and ZL contributed to the conceptualization and manuscript revision. All authors contributed to the article and approved the submitted version.

## Conflict of Interest

The authors declare that the research was conducted in the absence of any commercial or financial relationships that could be construed as a potential conflict of interest.

## Footnotes

1 http://www.mirbase.org/textsearch.shtml?q=virus
